# The Landscape of Protein Biomarkers Proposed for Periodontal Disease: Markers with Functional Meaning

**DOI:** 10.1155/2014/569632

**Published:** 2014-06-26

**Authors:** Nuno Rosa, Maria José Correia, Joel P. Arrais, Nuno Costa, José Luís Oliveira, Marlene Barros

**Affiliations:** ^1^Department of Health Sciences, Institute of Health Sciences (ICS), Center for Interdisciplinary Research in Health (CIIS), The Catholic University of Portugal, 3504-505 Viseu, Portugal; ^2^Department of Informatics Engineering (DEI), Centre for Informatics and Systems of the University of Coimbra (CISUC), University of Coimbra, 3030-290 Coimbra, Portugal; ^3^Department of Electronics, Telecommunications and Informatics (DETI), Institute of Electronics and Telematics Engineering of Aveiro (IEETA), University of Aveiro, 3810-193 Aveiro, Portugal

## Abstract

Periodontal disease (PD) is characterized by a deregulated inflammatory response which fails to resolve, activating bone resorption. The identification of the proteomes associated with PD has fuelled biomarker proposals; nevertheless, many questions remain. Biomarker selection should favour molecules representing an event which occurs throughout the disease progress. The analysis of proteome results and the information available for each protein, including its functional role, was accomplished using the OralOme database. The integrated analysis of this information ascertains if the suggested proteins reflect the cell and/or molecular mechanisms underlying the different forms of periodontal disease. The evaluation of the proteins present/absent or with very different concentrations in the proteome of each disease state was used for the identification of the mechanisms shared by different PD variants or specific to such state. The information presented is relevant for the adequate design of biomarker panels for PD. Furthermore, it will open new perspectives and help envisage future studies targeted to unveil the functional role of specific proteins and help clarify the deregulation process in the PD inflammatory response.

## 1. Introduction

The different forms of periodontal disease (PD) share four stages: (i) bacterial biofilm presence and accumulation in the gingival sulcus (colonization), (ii) bacterial penetration of epithelium and connective tissue in the gingiva adjacent to the tooth surface (invasion), (iii) stimulation of a host response involving activation of the innate and acquired immune response (inflammation), and (iv) irreversible destruction of connective tissue attachment to the tooth surface and bone (tissue loss) [1].

Gingival epithelial cells and fibroblasts in PD respond to Gram-negative bacterial lipopolysaccharide (LPS) by the transient expression of cytokines playing an active role in the initiation and maintenance of gingival inflammation [2]. The next line of defence comes with neutrophils that, under microbiota continuous stimulation, exhibit prosurvival and hyperresponsive behaviours [3, 4]. Periodontitis is also characterized by abundant monocyte infiltration, which finds the adequate signalling microenvironment to rapidly differentiate into macrophages, namely, through surface toll-like receptors [5]. Like neutrophils, macrophages phagocytose periodontal pathogens and additionally orchestrate wound repair by functionally coordinating innate and adaptive immune responses. This is achieved by the production of specific cytokines and chemokines which contribute to the attraction and activation of subsets of T cells. Of the multiple types of CD4+ T cells (Th1 Th2, Th17, and Tregs), Th2 cells are thought to dominate over an initial Th1 response in progressive periodontitis [6]. The different T cell subsets may participate in osteoclastogenesis process through RANKL production or the expression of another osteoclastogenic cytokine, the IL-17, accomplished by Th17 cells [7]. In spite of this knowledge, there is not solid evidence on the specific role of T cell subsets in periodontitis and the signalling process to activate or regulate them, with the exception that CD4+ T cells are induced by* P. gingivalis* to express RANKL [8].

Tregs are present in periodontal tissue [9] and this T cell subset is known for their anti-inflammatory role; however, the reasons for the apparent lack of anti-inflammatory function in PD are not clear.

B cells dominate chronic periodontitis lesions [10] and their activation and differentiation depend on T cells. The B cells spearhead multiple host defence mechanisms, including the production of antibodies, and in addition secrete a proinflammatory cytokine profile [11]. The role for B cell cytokines in oral pathogenesis is not clear, in part because other cell types also secrete the same B cell cytokines. However, it is well established that B cells are probably the major source of proosteoclastogenic RANKL [12].

A less obvious player in the innate immune response is the platelets, but, presently, the important role of these molecules has been acknowledged. Platelet toll-like receptor expression enables activated platelets to bind and capture bacteria. Subsequently, the platelets may directly kill the bacteria or aggregate around them and “trap” the bacteria for elimination by “professional” phagocytes. It is now clear that different subsets of platelets exist and that they can also heterotypically interact with a wide variety of immune system cells, including leukocytes [13]. However, the net results of these interactions are not clearly established in PD.

Despite abundant information and data in the literature regarding biomarkers for periodontal disease, we are still lacking suitable molecular markers of soft and hard tissue destruction which can replace the clinical gold standards [14]. In this review, we summarize, for each protein proposed as a biomarker elsewhere, the inferred functions of that protein in PD by comparing its role in another osteoimmunology processes. The pertinence of the proposal of each protein as a biomarker will be analyzed considering preferentially their exclusive presence in each PD variant, the quantification data, and the relevance of the molecular event represented by that protein in the context of PD.

## 2. Biomarker Survey in Periodontal Diseases

In order to obtain the list of proteins suggested as biomarkers in periodontal diseases, we queried the OralOme database using OralCard [15, 16] (http://bioinformatics.ua.pt/oralcard/) for the mesh terms “aggressive periodontitis,” “chronic periodontitis,” “gingivitis,” and “periimplantitis.” Each of the identified proteins was checked to confirm if it was suggested as a biomarker for periodontal diseases proteins > action view > biomarker (true/false) and the information for each protein quantification and presence in the different PD variants was also obtained.

To complement and update the information obtained from OralCard, a thorough manual bibliography review was performed in PubMed, to gather the most recent studies on proteins identified in PD. The following PubMed query was performed: (“proteomics” or “proteomic” or “proteome”) and (“saliva” title/abstract or “oral” title/abstract). The articles retrieved were manually scrutinized and key information from the proteins found in the various references was annotated as described before [16]. Briefly, for each protein, the following data were recorded when available: the identification of the protein; the source of the sample; the disease (MeSH code) and the up-/downregulation (fold change calculations performed according to Chan et al. [17]) compared to normal samples; sample donor data (age, gender, and social habits); methods of sampling and analysis; type of study (proteomics and nonproteomics); and posttranslation modifications and whether the protein had been proposed as a biomarker. Finally, all of these data are mapped to the PubMed citation where they were published. The data collected in this study were used to update the OralCard database.

To analyse and discuss PD biomarkers they were divided into two categories: quantified and nonquantified, depending on whether there was an indication of change in a disease state relative to healthy subjects (regulation). The proteins referred to as being increased (+) or decreased (−), without providing a value for this variation, were included in the group of nonquantified biomarkers. When a protein presented several regulation values from different studies, the average of these values was estimated.

Heterogeneity has become widely acknowledged as a phenomenon that is characteristic of pathogenesis [18, 19]. Heterogeneity occurs at two levels: as multiple phenotypes within an individual (patient-specimen heterogeneity) and between individuals (disease heterogeneity). In PD, both levels of heterogeneity are present. Therefore, we considered, as being differently present, proteins whose quantification had a fold change ≥3x (up- or downregulated) between health and disease samples. The quantified biomarkers were distributed in two groups: those with up-/downregulation ≥3x or counterregulated in different variants of PD and those with up- or downregulation <3x. The total set of biomarkers was analysed, annotating to which disease variant they are proposed for.

From the 43 proteins proposed as biomarkers for PD, only 8 were identified as being exclusively present in one of the disease variants (Table 1).

## 3. Biomarker Functional Analysis

The functional analysis of the biomarkers identified in the PD was performed using the statistical enrichment analysis feature from STRING [20]. Briefly, the list of all biomarkers identified in periodontal diseases was uploaded to STRING using UniProtKB AC codes and the protein network was built. Then, the statistical enrichment analysis feature was used to automatically detect statistically enriched molecular functions or biological processes in the network. For each biomarker, the associated gene ontology terms (GO molecular function and GO biological process) considered of interest in the physiopathological context of PD were annotated. Furthermore, for each biomarker, the GO cellular components terms were also included in the analysis. The OralCard tool was used to verify which periodontal biomarkers were identified in saliva samples in different studies and if they were suggested as biomarkers in nonperiodontal diseases.

All of this information was used to produce Table 2 and the proposed biomarkers are organized according to quantification in each disease; the disease variant and the type of sample in which they were identified; relevant biological processes in which they participate; their molecular functions; and cellular components where they are found.

The protein information presented, unless otherwise stated, was obtained from and is publicly available in the OralCard database.

Due to their pertinence as biomarkers, the proteins for which the up- or downregulation was ≥3x different from the health samples were discussed first.

## 4. The Functional Role of the Proteins Exclusively Proposed as Biomarkers for Chronic Periodontitis (CP)

In chronic periodontitis, 7 biomarkers exclusive to the pathology are suggested, but only 2, a chemokine (C-C motif) ligand 13 (Q99616), 4.97x upregulated in disease samples, and thymidine phosphorylase (P19971), 3.7x downregulated in disease samples, fulfill both requisites proposed for a biomarker, to be exclusive of the disease state and to have an up/down amount ≥3x.

The chemokine (C-C motif) ligand 13 (CCL13) is expressed in nonlymphoid tissues during chronic inflammation and acts as a chemotactic factor to attract monocytes, but not neutrophils, in tissues chronically exposed to exogenous pathogens. In rheumatoid arthritis, another disease involving inflammatory response, CCL13 was described as being associated with disease progression due to the increased macrophage infiltration and its antiapoptotic functions [21]. In CP, macrophage recruitment and antiapoptotic actions contribute to the permanence of these cells in periodontal tissues, a characteristic shared with other chronic inflammatory pathologies. Up to now, CCL13 was only found in crevicular fluid samples (Table 2); however, since this is an extracellular protein, it is possible to direct studies for its identification/quantification in saliva.

Thymidine phosphorylase has highly restricted target cell specificity, acting only on endothelial cells, promoting growth, angiogenic, and chemotactic activities. It is a cytosolic enzyme that is stored in platelets as a 45 kD single polypeptide chain and its presence in saliva has been detected. Thymidine phosphorylase was identified in mouth neoplasms where it is upregulated and promotes angiogenesis [22]. Because thymidine phosphorylase is downregulated in CP, the integrity of endothelial cells is compromised and it is possible that the normal mechanisms of angiogenesis are affected, leading to cellular hypoxia, characteristic of chronic inflammation and also linked to the promotion of anaerobic microbial growth.

The other two exclusive proteins suggested as biomarkers for CP have up/down quantification values <3x between health and disease samples: fibronectin (P02751), upregulated 1.84x, and the C-C motif chemokine 2 (P13500), upregulated 1.9x.

Fibronectin is an extracellular matrix protein involved in biological processes such as leucocyte migration and platelet degranulation. This protein, in spite of having been evaluated in CP, is a constitutive protein with low quantification values and therefore is not a promising biomarker candidate.

The C-C motif chemokine 2 is a chemotactic factor that attracts monocytes but not neutrophils [23]. This biomarker, which is upregulated only 1.9x, identifies the same process as the C-C motif chemokine ligand 13, which is upregulated 4.97x, and is considered more specific for the recruitment of monocytes in chronic situations.

Finally, 3 other proteins exclusive of PD were proposed as biomarkers but with no quantification (Table 2): angiotensinogen/serpin A8 (P01019) and clusterin (P10909) are both downregulated, while prostaglandin G/H synthase 2 (P35354) has only been identified as being present in CP.

Angiotensinogen/serpin A8 is cleaved by the enzyme renin and the resulting product, angiotensin I, is then processed by angiotensin converting enzyme (ACE) to generate the angiotensin II, the major effector peptide of renin-angiotensin system that mediates several key events of the inflammatory processes; namely, it increases vascular permeability via the release of prostaglandins [24]. It is in fact expected that angiotensinogen/serpin A8 is diminished in CP because it has been converted to angiotensin II and in this form will contribute to the increase of vascular permeability in this PD variant.

Clusterin is a 75–80 kDa disulphide-linked heterodimeric associated with the clearance of cellular debris and has been ascribed a plethora of functions such as recruitment, complement attack prevention, inhibition, and/or scavenging and inhibition [25]. Having clusterin in lower concentrations in CP may contribute to the lack of apoptotic induction of macrophages allowing them to remain in the tissues and consequently maintain a proinflammatory situation.

Prostaglandin G/H synthase 2 (COX-2) has a role as a major mediator of inflammation and is generally expressed only in cells which are upregulated during inflammation.

The clear role of COX-2 and angiotensin II as proteins is important in the inflammatory process and the fact that clusterin acts in several aspects of the immune response suggests the need for the quantification of these proteins in future studies of PD. COX-2 has been identified in crevicular fluid and mucosa samples, and angiotensin II and clusterin were found only in crevicular fluid; nevertheless, because the latter are extracellular, it may also be possible and useful to quantify them in saliva samples.

From the total of 7 exclusive proteins proposed as biomarkers in CP, chemokine (C-C motif) ligand 13 (upregulated 4.97x), characterizing the monocyte recruitment and differentiation stages, and thymidine phosphorylase (downregulated 3.7x), identifying angiogenesis involvement, are the two proteins which better identify CP.

## 5. A Panel for CP Biomarkers Using Proteins Which Are Common to Other PDs

Because most of the proteins proposed as biomarkers in PD are shared by disease variants (Table 2), it will not be possible to associate the majority of proposed biomarkers with specific events in each pathology. However, if the quantification of the protein is different enough and especially if the protein is counterregulated (upregulated in one pathology and downregulated in the other one), it may be a marker for a common molecular event, allowing the inference from the quantification data (up- or downregulated) of the role that protein may have in the CP.

The two major forms of periodontitis, chronic (CP) and aggressive (AP), do not display sufficiently distinct microbiological/immunological features and a recent work revealed limited differences between the gingival tissue transcriptional profiles of AP and CP, of genes related to immune response and apoptosis. Only signal transduction is overexpressed in AP, and genes related to epithelial integrity and metabolism are overexpressed in CP [26].

Table 2 presents 6 proteins present in CP and in other PDs, upregulated 3x relative to health: oncostatin-M (P13725), C-C motif chemokine 3 (P10147), protein S100-A9 (P06702), protein S100-A8 (P05109), azurocidin (P20160), and MMP9 (P14780).

Oncostatin-M is a multipotent cytokine produced by macrophages and activated T cells; it is structurally and functionally related to the IL-6 cytokine family and elevated expression levels of this cytokine have been determined in many inflammatory diseases [27, 28]. It seems that oncostatin-M alone, or in concert with proinflammatory cytokines like IL-1, can stimulate expression of genes that promote inflammation, namely, IL-6 [29], and also enhance the differentiation and proliferation of osteoblasts, inducing the formation of osteoclasts and consequently bone erosion [28, 30, 31]. Li et al. demonstrated that oncostatin-M can regulate MMP and TIMP mRNA in primary chondrocytes by activation of the Jak/Stat pathway [32] contributing to the expression of proteins involved in bone remodelling.

Oncostatin-M is upregulated 726x in CP and 128x in G. This protein was proposed as a biomarker for both CP and G (Table 2). Considering its role in the modulation of inflammatory processes, it is recommended that further quantification studies are designed to determine the levels of protein present in each PD. If the quantification results are promising, this protein can be a biomarker to predict the evolution from G to a CP status.

The C-C motif chemokine 3 or MIP-1 alpha (macrophage inflammatory protein 1 alpha) is a member of the CC or beta chemokine subfamily that binds to CC chemokine receptor 1 (CCR1) and CCR5 with high affinity. CC chemokines mainly act on monocytes and lymphocytes without affecting neutrophils [33]. In rheumatoid arthritis patients, the C-C motif chemokine 3 is associated with disease progression as a result of its antiapoptotic effects, increased macrophage infiltration, and synovial tissue angiogenesis [34]. C-C motif chemokine 3 in CP is upregulated 18x which contributes to macrophage presence due to the antiapoptotic effect of this protein and therefore promotes the establishment of a chronic inflammatory response. C-C motif chemokine 3 has also been identified in samples from periimplantitis patients but there are no published quantification data for this clinical situation.

S100 protein family is a prominent player in innate immunity [35]. Two S100 proteins, namely, protein S100-A9 and protein S100-A8, are calcium- and zinc-binding proteins abundant in the cytosol of neutrophils [36] which are released as a heterodimeric complex, S100A8/A9, under inflammatory conditions [37]. As a calprotectin (S100A8/A9) complex, it has a wide array of intra- and extracellular functions and recently has been associated with numerous human disorders, including acute and chronic inflammatory conditions [38–42]. Apparently, these proteins are able to perform cytokine-like and chemokine-like roles via activation of toll-like receptor 4 (TLR4) dependent signalling cascades and potentially other signalling pathways. Binding to TLR4 activates the MAPK and nuclear factor NF-kappa-B signalling pathways resulting in the amplification of the proinflammatory cascade [43].

Protein S100-A9 is upregulated in CP 10.4x, downregulated in AP 1.4x, and downregulated in G 1.75x, whereas protein S100-A8 is upregulated 3.07x in CP. The calprotectin complex S100-A9/S100-A8 may act as the main trigger for the activation of the proinflammatory cascade in CP.

Azurocidin was proposed as a biomarker in CP and G. This protein is upregulated in CP 6.5x and 3.35x in G. Azurocidin is a chemokine produced by neutrophils that attracts monocytes in a second wave of invasion [44], which clearly reflects the synergy of mechanisms, induced by the recurring microbial challenge characteristic of CP and G that contribute to the permanence of macrophages in periodontal tissues.

Matrix metalloproteinases are considered modifiers of host response and it has been suggested that their role and involvement should be interpreted not solely as surrogate promoters of tissue destruction, but also as defensive or protective factors against inflammation as a whole [45, 46]. MMPs produced by inflammatory cells, such as macrophages and neutrophils, facilitate migration and recruitment of cells, including inflammatory cells, and are also related to angiogenesis and vascular remodelling [47, 48].

Matrix metalloproteinase-9 plays an essential role in leukocyte migration by cleaving the IL-8 precursors, but many other cytokines are also substrates, including TNF-*α* and interleukin-1*β* [49]. On the other hand, proinflammatory cytokines are implicated in the transcriptional control of MMP-9 [50, 51]. MMP-9 is upregulated 3.54x in CP; furthermore, it is also annotated as upregulated in G but without quantification. In PI this protein is only reported as present without any quantification or regulation data.

Among the proteins proposed as biomarkers for CP, there are eight which are common to other PDs but have quantification values up/down <3x when compared to health, which makes them less adequate as biomarkers: prolactin-inducible protein (P12273), superoxide dismutase (P00441), lactotransferrin (P02788), peptidyl-prolyl* cis*-*trans* isomerase A (P62937), apolipoprotein A-I (P02647), cystatin-SN (P01037), mucin 5B (Q9HC84), and cystatin-S (P01036). Of these, prolactin-inducible protein may act on different subsets of T cells via the CD4 receptor and have antiapoptotic action. The fact that this protein is downregulated (2.55x) may denote the involvement in the mechanism by which, for example, Treg lymphocytes are eliminated in chronic inflammatory processes. Further studies are recommended to evaluate the role of this protein in PD.

## 6. Functional Roles of the Main Proteins Common to AP and CP: Are There Biomarkers for AP?

From the analysis of PD proteomes and the integration of the information on the proteins proposed as biomarkers for AP (Table 2), a set of 14 proteins seems to be the most promising considering their quantification data, but all proteins were identified in other variants of PD (Table 2). Even though some of the 14 proteins have not been proposed earlier as biomarkers for AP, the quantification data suggest that they might be an adequate choice. The following proteins ordered by decreasing fold change relative to the quantification in AP and health: hemoglobin subunit delta (P02042), RANKL (O14788), osteoprotegerin (O00300), neutrophil gelatinase-associated lipocalin (P80188), plastin (P13796), neutrophil defensin 1 (P59665), serotransferrin (P02787), lysozyme C (P61626), annexin A1 (P04083), profilin-1 (P07737), Ig alpha-1 chain C region (P01876), Ig gamma-2 chain C region (P01859), Ig alpha-2 chain C region (P01877), and dermcidin (P81605). All of these proteins have been previously identified in saliva except dermcidin identified only in crevicular fluid.

One of the hallmarks of periodontitis has been the massive accumulation of neutrophils, which can be found in the gingival connective tissue, the junctional epithelium, and especially the periodontal pocket, where they constitute the overwhelming majority of recruited leukocytes [52]. Among the proteins excreted by activated neutrophils, neutrophil gelatinase-associated lipocalin (NGAL) is released from azurophil, specific, and gelatinase granules [53]. NGAL is an important protein protecting against Gram-negative bacterial infection and is a ligand for MMP-9 activating this enzyme. The expression of NGAL is altered in inflammatory conditions [54]. Agents that induce NF-*κ*B signalling, such as IL-1*β* and TLRs activation via LPS, positively regulate NGAL expression. Additionally a cross-talk between the JNK (activated via LPS) and NF-*κ*B has been suggested to act together to upregulate NGAL expression [55]. NGAL is upregulated 18.9x in AP, while in CP it is upregulated only 1.8x. The increased expression of NGAL in AP may be one of the few effective antimicrobial mechanisms since most of the proteins involved in microbial defence are downregulated.

Defensins are small, cationic, antimicrobial peptides that are considered to be important antibiotic-like effectors of innate immunity. Defensins also function as immunomodulatory molecules by inducing cytokine and chemokine production, as well as inflammatory and immune cell activation [56]. By using chemokine receptors on dendritic cells and naïve T cells, defensins might also contribute to the regulation of host adaptive immunity. Recently, the role of neutrophil defensin 1 in the integrity of keratinocytes has been demonstrated and in low concentrations of this molecule, keratinocyte proliferation is enhanced [57]. Neutrophil defensin 1 is one of the counterregulated proteins; it is 14.8x downregulated in AP and 4x upregulated in CP. Up to now, there is no evidence available on the exact role of neutrophil defensin 1 in AP. Is the decrease in neutrophil defensin 1 responsible for the apparent decrease in the host's capacity to fight off infection characteristic of AP? Or is the decrease in this molecule aimed only at guaranteeing the proliferation of the keratinocytes to compensate tissue destruction characteristic of PD?

Another protein released by neutrophils, annexin I, inhibits the signal transduction pathway, acting as a negative regulator of proinflammatory mediators including IL-1 and IL-6. Annexin I also regulates phospholipase A2 activity and consequently the cyclooxygenase-2. Annexin I has the ability to restrict leukocyte transmigration and recruitment during inflammation in a MAPK-dependent manner [58–60]. In several experimental models of arthritis, absence of annexin-1 has been associated with increased levels of cytokines and exacerbation of acute inflammation [61, 62]. Annexin A1 is downregulated 8.1x in AP and upregulated 1.5x in CP which means that a low concentration of this protein in AP contributes to a higher neutrophil migration and an increase in proinflammatory molecules.

Plastin-2 is a leukocyte-specific F-actin-bundling protein implicated in cell migration, neutrophil function, DNA repair, and endocytosis [63, 64]. The expression of plastin-2 is restricted to leukocytes, although L-plastin is also aberrantly upregulated in many cancer cells [63–65]. A recent study demonstrates an important role for plastin-2 and the signalling pathways regulating its phosphorylation in response to chemokines which adds plastin-2 to a growing list of proteins implicated in T lymphocyte polarity and migration [66]. Plastin-2 is upregulated 15.6x in AP and 4.9x in CP and is probably one of the proteins responsible for the migration and activation of T cells in PD. This protein has been proposed as a biomarker for AP, CP, and G.

The role of the receptor activator of nuclear factor NF-kappa-B ligand and the osteoprotegerin (RANKL-OPG), a bimolecular system considered as the “bottle-neck” regulator of osteoclastogenesis and bone resorption, in both physiological and pathological conditions, is well known. However, the sequence of molecular and cellular events which lead to the production of these molecules in PD is not clear. Neutrophils can induce osteoclastic bone resorption through the expression of membrane-bound RANKL but could only mediate periodontal bone resorption if they are in close proximity to the bone [67]. Nevertheless, all resident mesenchymal cells may also express RANKL under bacterial challenge [68]. It was recently stated that the increased RANKL/OPG ratio may denote the occurrence of periodontitis but may not predict ongoing disease activity because its steadily elevated levels frequently remain after treatment, suggesting that the molecular mechanisms of bone resorption are still active [69]. RANKL is upregulated 57x in AP, 16x in CP, and 12x in PI. Osteoprotegerin (OPG) is downregulated 4x in AP, 2.28x in CP, and 2.44x in PI.

Together these 6 proteins, neutrophil gelatinase-associated lipocalin, neutrophil defensin 1, annexin A1, plastin, RANKL,and OPG, identify the following molecular mechanisms present in AP: (i) high number of neutrophils remaining in the tissue which leads to an exaggerated innate immune response and prevalence of the associated molecular mechanisms (neutrophil gelatinase-associated lipocalin); (ii) decrease in the antimicrobial defence mechanisms (neutrophil defensin 1); (iii) increase in the expression of proinflammatory cytokines due to a decrease in the concentration of the main anti-inflammatory molecules (annexin A1); (iv) the onset of the acquired immune response by the recruitment and activation of T lymphocytes (plastin); (v) strong activation of osteoclastogenesis by a great increase in the protein regulating the differentiation and activation of osteoclasts (RANKL) accompanied by a corresponding decrease in the OPG.

Neutrophil gelatinase-associated lipocalin and neutrophil defensin 1 have not been suggested as biomarkers for AP yet, only for CP (Table 2). However, from the functional role and the quantification data, we suggest that these two proteins are the biomarkers which identify AP more specifically.

From the remaining proteins in the proposed biomarker group, 3 act as antimicrobial molecules and are downregulated in AP and upregulated in CP revealing that the general antimicrobial defence mechanisms are definitely compromised.

Dermcidin forms tiny channels perforating the cell membrane and its presence in granules of neutrophils has been reported [53]. It is also known that dermcidin expression is not induced under inflammatory conditions in keratinocytes [70, 71]. In AP, dermcidin is downregulated 2.3x and it is upregulated 1.4x in CP.

Lysozyme C is a proteolytic enzyme secreted by oral leucocytes that acts on both peptide-substituted and unsubstituted peptidoglycan. Lysozyme C is downregulated 9.1x in AP, upregulated 1.99x in CP, and downregulated 1.41x in G.

Serotransferrin binds iron and prevents bacterial survival. The level of serotransferrin decreases in inflammation [72]. Serotransferrin is downregulated 10.4x in AP and upregulated 1.56x in CP.

Another protein present in AP proteome is profilin-1, an actin-binding protein, which is widely distributed in various types of cells with highly conserved sequences. Profilin-1 achieves its function via the regulation of unpolymerised actin in cells contributing to endothelial cell contraction and vascular hyperpermeability. Recently, it has been reported that overexpression of profilin-1 upregulated the expression of ICAM-1, increased endothelial cell permeability, induced endothelial cell apoptosis, and promoted endothelial cell migration [73]. Under pathological conditions, such as diabetes or atherosclerosis, profilin-1 levels increase. It has been verified that AGEs upregulated the expression of profilin-1 via the excess production of ROS and subsequent activation of PKC and NF-*κ*B [73].

Profilin 1 is upregulated in AP 4.5x, in CP 2.51x, and 2.00x in G. This protein has been proposed as a biomarker only for CP. Due to this protein's relevance in vascular tissue lesion, further studies in PD are recommended as it may be an early indicator of the host's response in PD and will be the link between PD and cardiovascular disease.

Finally, keratin type II cytoskeletal 1 (P04264) is downregulated 44.3x in AP and upregulated 34.9x in CP. Keratin, type II cytoskeletal 2 oral, is downregulated 6.4x in AP and upregulated 5.2x in CP. McLaughlin demonstrated that the keratin concentration in GCF was significantly higher at sites exhibiting signs of gingivitis and periodontitis compared with healthy sites [74]. The OralCard data shows counterregulated quantities of keratins in AP versus CP. The keratins are the typical intermediate filament proteins of epithelia, showing an outstanding degree of molecular diversity. They are expressed in highly specific patterns related to the epithelial type and stage of cellular differentiation. The keratin, type II cytoskeletal 2 oral, is synthesized during maturation of epidermal keratinocytes. Stress conditions affect not only keratin expression profiles, but also keratin expression levels and posttranslational modification. In terms of the importance of keratins for PD diagnostics there is not enough evidence to associate the differential expression present in AP and CP.

Because we have not identified a single protein exclusive of AP, we consider that, with the functional information available, the conjugation of neutrophil gelatinase-associated lipocalin upregulated in AP, with neutrophil defensin 1 and annexin A1 both downregulated in AP, may be used to identify AP.

Belibasakis and Bostanci suggest studies establishing the levels of RANKL to discriminate between health and periodontal disease [69]. These results are important to establish the RANKL level that discriminate between health and the PD variants.

Serotransferrin, a keratin type II cytoskeletal 1, and keratin type II cytoskeletal 2 oral, being counterregulated in AP and CP, may allow the differential diagnostic between the two pathologies.

## 7. The Scenario of the Proposed Biomarkers for Perimplantitis

The sequence of immunopathological events and the qualitative composition of the immune cells in periimplant infections are similar to that of periodontal infections. Nevertheless, compared to periodontitis, periimplantitis is marked by a more extensive inflammatory infiltrate and innate immune response, a greater severity of tissue destruction, and a faster progression rate [75].

In PI, a single exclusive protein has been proposed as a biomarker: alkaline phosphatase, tissue-nonspecific isozyme (P05186) which is upregulated 5.9x.

Alkaline phosphatase (TNAP) belongs to a ubiquitous family of metalloenzymes that play an essential physiological role during osteoblastic bone matrix mineralization [76, 77]. It is expressed on the cell membrane of osteoblasts and odontoblasts, as an ectoenzyme transported to the plasma membrane. Infections, including bone infections, can lead to increased tissue-nonspecific alkaline phosphatase levels. TNAP is also present in neutrophils granules and its activity increases in bacterial infection [78]. As to the relevance of this enzyme in PD, the literature is not consensual; some authors state that elevated alkaline phosphatase levels precede attachment loss, when the clinical parameters are not yet discriminatory [79], and others consider that there is no support for the predictive value of alkaline phosphatase levels in periodontal breakdown but that it may serve as a marker in periodontal treatment planning and monitoring [80, 81]. Another function of TNAP is its ability to remove one of the two phosphate groups from the lipid moiety of the Gram-negative bacterial LPS, which results in the formation of nontoxic dephosphorylated monophosphoryl LPS (MPLPS), ineffective to activate the TLR receptors [82]. Two other proteins proposed as biomarkers in PI were also identified: neutrophil collagenase/MMP-8 (P22894) upregulated 1.1x in AP, 1.82x in CP, and 9.7x in PI and neutrophil elastase (P08246) upregulated 2x in AP, 6.5x in CP, and 12.8x in PI.

MMP-8, released from neutrophils and potentially other cellular sources at sites of inflammation, activates IL-8 and IL-6 and chemokines, creating a “feed-forward” response that drives further neutrophil recruitment and a self-reinforcing protease-immunomodulatory circuit that may underpin its function in diverse physiological repair and defence mechanisms [83, 84].

Neutrophil elastase (NE), a cationic glycoprotein, is stored in readily active form in PMN primary granules [85], is a key antimicrobial enzyme that mediates defence against Gram-negative bacteria by degradation of structural proteins localized on the cell wall [86] or by targeting their virulence factors [87]. NE also binds to PMN-derived chromatin structures, termed neutrophil extracellular traps, and exerts its own antimicrobial function [88].

Alpha-2-macroglobulin (P01023) (*α*2-M), one of the acute phase proteins, apart from inhibiting proteinases, regulates binding of transferrin to its surface receptor, binds defensin and several important cytokines, including interleukin-1*β* and interleukin-6, and modifies their biological activity [89]. Alpha-2-macroglobulin is upregulated 2.15x in CP and 8.1x in PI, but it has been proposed as a biomarker only in CP.

For the 4 proteins which represent a greater impact on PI, it is still not possible to clarify their participation in PD, but considering their role, namely, as immune response modulators, a deeper study of their influence in PD is justified.

## 8. The Proposed Biomarkers in Gingivitis

Although dental plaque accumulation causes gingivitis (a reversible form of periodontal inflammation that does not involve the alveolar bone), gingivitis in turn does not necessarily lead to periodontitis, suggesting that stable gingivitis means that a protective host response exists.

As biomarkers for G, the following molecules were proposed: oncostatin-M and azurocidin, already discussed in CP; RANKL which is shared by all PD variants being the lower values observed in G; and plastin-2 and serotransferrin shared with AP and CP, which have been proposed as biomarkers in G but have not been quantified in this disease variant.

Our analysis points to neutrophil defensin 3 as a molecule which may be suitable for the differential diagnostic between G and CP. In addition to its antimicrobial effects, it has also been reported to attract monocytes [90] and T and dendritic cells [91]. Neutrophil defensin 3 is counterregulated in G and CP, and future studies should be performed to evaluate if the protein is present in quantities up/down 3x.

## 9. Conclusions

**Figure 1:**
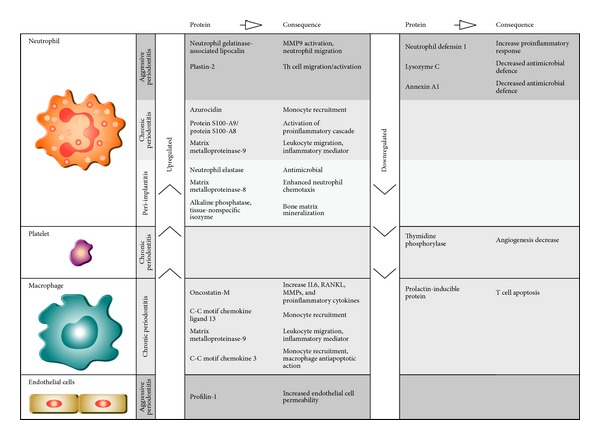
Biomarkers selected by their quantification to characterize each PD variant. The cells which produce the different proteins are identified. For each protein, the consequences of the modification in the functional role, inducing their up- or downregulation, within the host inflammatory response, are suggested.

The analysis of the 43 proteins proposed as biomarkers for PD allowed the identification of their functional role and the cells which produce them.  Figure 1 shows a selection of biomarkers that, due to their quantification, better characterize each PD variant. Clearly, the biomarkers proposed for CP are produced by neutrophils and macrophages and are involved in mechanisms responsible for monocyte and neutrophil recruitment and the regulation of proinflammatory molecule production. Platelets are a source of thymidine phosphorylase, a protein eventually implicated in the deregulation of angiogenesis.

In contrast, the biomarkers proposed for AP are essentially produced by neutrophils. The quantification data of these proteins points to a decrease in antimicrobial defence mechanisms.

The biomarkers proposed for PI are also produced by neutrophils. They represent mechanisms involved in the innate response and bone remodelling processes.

Some of the biomarkers proposed so far for PD are promising but the quantification data are scarce. Further quantitative proteomic studies are needed not only to establish the expression levels of these proteins in each PD variant, but also to establish the protein levels associated with health.

## Figures and Tables

**Table 1:** Quantification of the biomarkers proposed for each disease variant of PD.

Periodontal disease	Proposed biomarker	Biomarker with quantification	Biomarker up/down	Exclusive biomarker
Aggressive periodontitis	7	7	0/0	0
Chronic periodontitis	37	34	2/5	7
Gingivitis	9	7	0/0	0
Periimplantitis	3	3	1/0	1

**Table 2:** Information used for the functional analysis and critical appraisal of each protein suggested as biomarker.

Protein name	UniprotKB AC	Gene name (STRING)	Disease and disease biomarker	Quantification (fold change)	Saliva (OralCard)	CF (OralCard)	Mucosa (OralCard)	Saliva non-PD (OralCard)	Extracellular (GO CC)	Intracellular (GO CC)	Intracellular membrane bonded (GO CC)	Secretory granule (GO CC)	Endocytic vesicle (GO CC)	Immune system process (GO BP)	Regulation of immune system process (GO BP)	Innate immune response (GO BP)	Response to bacterium (GO BP)	Response to LPS (GO BP)	Leucocyte migration (GO BP)	Regulation of cytokine production (GO BP)	Inflammatory response (GO BP)	Regulation of inflammatory response (GO BP)	Positive regulation of acute inflammatory response (GO BP)	Response to interleukin 1 (GO BP)	Enzyme inhibitor activity (GO MF)	Positive regulation of MAPK cascade (GO BP)	Jak-Stat signaling (GO BP)	Regulation of ERK1/2 cascade (GO BP)	Adaptive response (GO BP)	T cell activation (GO BP)	Osteoclastogenesis (GO BP)	Coagulation/wounding (GO BP)	Platelet activation (GO BP)	Platelet degranulation (GO BP)	Platelet-derived growth factor receptor binding (GO MF)	Angiogenic activity (GO BP)	Regulation of peptidase activity (GO MF)	Ras GTPase binding activity (GO MF)	Biomarker for other pathologies (OralCard)
C-C motif chemokine ligand 13	Q99616	CCL13	PC	4.97		+			+										+		+																		+

Thymidine phosphorylase	P19971	TYMP	PC	−3.70		+		+		+																									+	+			+

C-C motif chemokine 2	P13500	CCL2	PC	1.90	+	+								+					+																				+

Fibronectin (FN)	P02751	FN1	PC	1.84	+	+		+						+					+													+		+					

Angiotensinogen (serpin A8)	P01019	AGT	PC	—		+		+	+	+										+		+				+													

Clusterin	P10909	CLU	PC	—		+		+	+			+		+	+	+													+			+	+	+					

Prostaglandin G/H synthase 2 (COX-2)	P35354	PTGS2	PC	0.00		+	+			+	+						+			+	+	+	+													+			+

Oncostatin-M	P13725	OSM	PC G	726.0 128.0		+			+													+	+			+	+												

C-C motif chemokine 3	P10147	CCL3	PC PI	18.00 +	+	+															+																		+

Protein S100-A9	P06702	S100A9	PA PC G	−1.40 10.41 −1.75	+	+		+	+	+						+	+	+	+	+						+		+											+

Protein S100-A8	P05109	S100A8	PC G	3.07 0.00	+	+		+	+	+							+	+																					+

Azurocidin	P20160	AZU1	PC G	6.15 3.35		+		+		+		+					+		+	+	+																		+

Matrix metalloproteinase-9	P14780	MMP9	PC G PI	3.54 0.00 +	+	+		+	+								+	+						+															+

Prolactin-inducible protein	P12273	PIP	PC G	−2.55 0.00	+	+		+						+																									+

Superoxide dismutase	P00441	SOD1	PA PC	0.00 1.85	+	+		+						+																		+		+					

Lactotransferrin	P02788	LTF	PA PC	0.60 1.94	+	+		+			+			+			+																						

Peptidyl-prolyl cis-trans isomerase A	P62937	PPIA	PC G	1.73 0.00	+	+		+						+	+				+													+		+					

Apolipoprotein A-I	P02647	APOA1	PC G	1.56 0.00		+		+							+																	+		+					

Cystatin-SN	P01037	CST1	PC G	−1.47 0.00	+	+		+																													+		+

Mucin 5B	Q9HC84	MUC5B	PC G	1.01 0.00	+			+	+	+	+																												

Cystatin-S	P01036	CST4	PC G	−0.10 0.00	+	+		+																													+		

Hemoglobin subunit delta	P02042	HBD	PA PC G	158.2 30.87 11.00		+		+																								+							+

RANKL	O14788	TNFSF11	PA PC G PI	56.99 16.09 1.32 12.89	+	+	+		+	+									+			+	+			+		+											

Osteoprotegerin	O00300	TNFRSF11B	PA PC G PI	−4.00 −2.28 −2.44 −0.71	+	+	+	+																							+								+

Neutrophil gelatinase-associated lipocalin	P80188	LCN2	PA PC	18.90 1.77	+	+		+	+	+						+	+	+						+															+

Plastin-2	P13796	LCP1	PA PC G	15.60 4.93 0.00	+	+		+	+	+																				+									+

Neutrophil defensin 1	P59665	DEFA1	PA PC	−14.8 4.00	+	+		+	+			+					+																						+

Serotransferrin	P02787	TF	PA PC G	−10.4 1.56 0.00	+	+		+		+	+		+																			+	+	+					+

Lysozyme C	P61626	LYZ	PA PC G	−9.10 1.99 −1.41	+	+		+	+								+				+																		+

Annexin A1	P04083	ANXA1	PA PC G	−8.10 1.46 −1.43	+	+		+	+	+											+	+		+	+														+

Profilin-1	P07737	PFN1	PA PC G	4.50 2.51 2.00	+	+		+		+	+																					+	+	+				+	+

Ig alpha-1 chain C region	P01876	IGHA1	PA PC G	−4.70 1.67 0.00	+	+		+																					+										+

Ig gamma-2 chain C region	P01859	IGHG2	PA PC	3.55 1.88	+	+		+																					+										+

Ig alpha-2 chain C region	P01877	IGHA2	PA PC	3.10 −1.19	+	+		+																					+										+

Dermcidin	P81605	DCD	PA PC G	−2.80 1.39 0.00		+											+																						+

Keratin, type II cytoskeletal 1	P04264	KRT1	PA PC G	−44.3 34.9 −1.45	+	+		+		+						+																							+

Keratin, type II cytoskeletal 2 oral	Q01546	KRT76	PA PC G	−6.4 5.18 −1.79		+		+		+																													

Complement C3	P01024	C3	PA PC	−1.30 1.58	+	+		+	+							+					+		+		+														+

Neutrophil elastase	P08246	ELANE	PA PC G PI	2.00 6.50 0.00 12.80		+		+		+		+					+	+	+	+	+	+																	+

Neutrophil collagenase	P22894	MMP8	PA PC PI	1.10 1.82 9.70	+	+		+	+										+																				+

Alpha-2-macroglobulin	P01023	A2M	PC G PI	2.15 0.00 8.10	+	+		+	+	+		+										+																	+

Alkaline phosphatase. tissue-nonspecific isozyme	P05186	ALPL	PI	5.90		+			+								+	+																					

Neutrophil defensin 3	P59666	DEFA3	PC G	2.63 −2.08	+	+		+									+																						+

GO CC: gene ontology cellular component; GO BP: gene ontology biological process; GO MF: gene ontology molecular function.

## Abbreviations

ACE: Angiotensin converting enzyme
AGEs: Advanced glycation end-products
AP: Aggressive periodontitis
CCL13: Chemokine (C-C motif) ligand 13
CCR1: CC chemokine receptor 1
CD4: T cell surface glycoprotein CD4
COX-2: Prostaglandin G/H synthase 2
CP: Chronic periodontitis
G: Gingivitis
ICAM-1: Intercellular adhesion molecule 1
IL-1: Interleukin-1
IL-17: Interleukin-17
IL-6: Interleukin-6
IL-8: Interleukin-8
Jak/Stat: Janus kinase/signal transducer and activator of transcription
JNK: c-Jun N-terminal kinase
LPS: Lipopolysaccharide
MAPK: Mitogen activated protein kinases
MIP-1: Macrophage inflammatory protein 1
MMP: Matrix metalloproteinase
MPLPS: Monophosphoryl lipopolysaccharide
NE: Neutrophil elastase
NF-*κ*-B: Nuclear factor NF-kappa-B
NGAL: Neutrophil gelatinase-associated lipocalin
OPG: Osteoprotegerin
PD: Periodontal diseases
PI: Periimplantitis
PKC: Protein kinase C
PMN: Polymorphonuclear leukocytes
RANKL: Tumor necrosis factor ligand superfamily member 11
ROS: Reactive oxygen species
STRING: Search tool for the retrieval of interacting genes/proteins
Th: T helper cells.
TIMP: Tissue inhibitor of metalloproteinases
TLR4: Toll-like receptor 4
TNAP: Tissue-nonspecific alkaline phosphatase
TNF-*α*: Tumor necrosis factor
Tregs: Regulatory T cells
*α*2-M: Alpha-2-macroglobulin.
